# Pectin from Fruit- and Berry-Juice Production by-Products: Determination of Physicochemical, Antioxidant and Rheological Properties

**DOI:** 10.3390/foods12081615

**Published:** 2023-04-11

**Authors:** Daiga Konrade, Sergejs Gaidukovs, Francisco Vilaplana, Pramod Sivan

**Affiliations:** 1 Institute of Technology of Organic Chemistry, Faculty of Materials Science and Applied Chemistry, Riga Technical University, P. Valdena Str. 3/7, LV-1048 Riga, Latvia; 2 Latvia Institute of Polymer Materials, Faculty of Materials Science and Applied Chemistry, Riga Technical University, P. Valdena Str. 3/7, LV-1048 Riga, Latvia; 3 Department of Chemistry, Division of Glycoscience, KTH Royal Institute of Technology, SE-100 44 Stockholm, Sweden

**Keywords:** antiradical scavenging activity, esterification, gels, monosaccharides, phenolics, rheology, viscosity

## Abstract

Plums (*Prunus domestica*)*;* red currants (*Ribes rubrum*)*;* black currants (*Ribes nigrum*)*;* gooseberries (*Ribes uva-crispa*)*;* sour cherries (*Prunus cerasus*); pumpkins (*Cuccurbita* spp.) are sources for valuable fruit- and berry-juice and cider production. This process leaves a large number of by-products (BP) in the form of pomace, which accounts for up to 80% of the raw material. This by-product represents a rich source of biologically active compounds, especially in the form of different pectic polysaccharides. The pectin extracted from commercial fruits such as citric fruits and apples has high medicinal properties, can be used as edible films and coatings, and is also useful in texture improvement and gel production in the food industry. However, many under-utilized fruits have received little attention regarding the extraction and characterization of their high/value pectin from their by-products. Moreover, the commercial extraction process involving strong acids and high temperature to obtain high-purity pectin leads to the loss of many bioactive components, and these lost components are often compensated for by the addition of synthetic antioxidants and colorants. The aim of the research is to extract pectin from juice production by-products with hot-water extraction using weak organic (0.1 N) citric acid, thus minimizing the impact on the environment. The yield of pectin (PY = 4.47–17.8% DM), galacturonic acid content (47.22–83.57 g 100^−1^), ash content (1.42–2.88 g 100 g^−1^), degree of esterification (DE = 45.16–64.06%), methoxyl content (ME = 4.27–8.13%), the total content of phenolic compounds (TPC = 2.076–4.668 µg mg^−1^, GAE) and the antiradical scavenging activity of the pectin samples (DPPH method (0.56–37.29%)) were determined. Free and total phenolic acids were quantified by saponification using high-pressure liquid chromatography (HPLC). The pectin contained phenolic acids—benzoic (0.25–0.92 µg mg^−1^), gallic (0.14–0.57 µg mg^−1^), coumaric (0.04 µg mg^−1^), and caffeic (0.03 µg mg^−1^). The pectin extracts from by-products showed glucose and galactose (3.89–21.72 g 100 g^−1^) as the main neutral sugar monosaccharides. Pectin analysis was performed using FT-IR, and the rheological properties of the pectin gels were determined. The quality of the obtained pectin from the fruit and berry by-products in terms of their high biological activity and high content of glucuronic acids indicated that the products have the potential to be used as natural ingredients in various food products and in pharmaceutical products.

## 1. Introduction

Pectin is a complex acid-rich polysaccharide from plant cell walls with important industrial application as a multifunctional and versatile hydrocolloid [1,2,3]. The molecular structure of pectin is formed of three main regions: homogalacturonan (HG), rhamnogalacturonan I (RG-I) and rhamnogalacturonan II (RG-II) [4]. The linear HG region is composed of α-(1,4)-linked D-galacturonic acid (Gal A) units that may be methyl-esterified at the C-6 carboxyl or acetylated at the O-2 and/or O-3 [2,4,5,6]. The structure and properties are important for the classification and functionality of pectin for food and cosmetic product design [3,7,8].

The yield and composition of pectin depend on the plant source, harvest time, extraction technique, and conditions employed during pectin isolation and purification [9,10,11,12]. Extensive research during the past several decades resulted in the development of different pectin extraction methods such as conventional heating in acid [13], enzymatic extraction [14], microwave [14,15,16,17], ultrasound [10], combined methods [18], electromagnetic and autoclaving [7,14,18,19,20,21,22]. Extraction of pectin with the conventional method is carried out with hot acidified water with inorganic: sulfuric, nitric, hydrochloric [23], or organic acids: tartaric, citric, (temperature above 60 °C; pH range of 1.5–3; time 0.5–6 h [14,24]. Usage of these methods can increase the degradation of pectin; the method is considered time-consuming and can cause irreversible environmental pollution [3]. To minimize the impact on the environment, researchers have found that pectin with higher molecular weight and viscosity can be obtained by using weak citric acid instead of mineral acid and the pectin extraction yields are higher than other methods [12,18,25,26].

By-products from the juice- and cider-production industry are promising sources of pectin that can be further used as food ingredients—E440a (pectin) and E440b (amidated pectin) [2,6]—and in non-food industries as a thickener, gelling ingredient [27,28], and cation-binding agent [29]. Furthermore, pectin is a source of antioxidants [30], as polysaccharides from fruits and berries have a chemical structure that provides anti-diabetic [31], immunomodulating [32], antitussive, and astringent biological properties [2,29,32,33,34]. The consumption of pectin impacts blood cholesterol levels [7]; it removes toxins from the body and it regulates blood glucose levels [13,29,35,36,37,38]. Pectin can form gels that can be used for biomedical applications, such as drug delivery, tissue engineering, and wound dressing [8,27,39].

Reducing food loss and waste in all fields of food production and consumption has gained increased attention on the part of society [40]. Food waste in primary food production is about 9.1 ± 1.5 MT, and 16.9 ± 2.7 MT in processing [41]; moreover, the fruit- and berry-juice industry is one of the largest agro-based industries and, therefore, by-products occur–peel, pomace, seeds, core, etc. [42]. Fruit and berry pomace after juice extraction contains up to 16% of the mass of fruits being purposed for processing [43,44,45]. By-products are still rich in biologically active substances such as fibres [46], vitamins [47], carotenes [48], organic acids [49], and macronutrients [50] that play an important role in human health [41,51,52,53].

Rhubarb (*Rheum rhabarbarum*) is early-spring raw material for juices, with an important economic value [54,55]. For beverages, the juice of the rhubarb is extracted with a cold press and, therefore, by-products such as pomace are left, and are still rich in bioactive compounds such as anthraquinones, stilbenes, flavonoids, and tannins [56]. Plums (*Prunus domestica*), after apples, pears and peaches, are the third most popular fruit grown in Europe, with an annual production of 27,700 tonnes in 2021, and account for about 25% of the whole world’s plum production. The chemical composition of plum fruits includes sugars, organic acids, tannins and dye extracts, pectin, vitamins and mineral salts [57,58]. Apples (*Malus domestica*) are widely grown, and are used in food manufacturing for juice, cider, wine, distilled spirit and vinegar manufacturing in EU countries [59] and solid waste represents 20 to 35% of the fresh weight of the apple fruit [45,60,61]. About 9.2–12.8% of pectic substances have been reported in the pomace from the juice industry [60,62]. The residues of by-products from fruits and berries consist of peel, core, seed, calyx, stem and pulp. Most pectin substances come from the epi-mesocarp, accounting for up to 95.5% of the solid waste [63,64]. Although the by-product production from promising fruits is very high, there is little known of its pectin content and application potential in various commercial purposes.

Therefore, the aim of the research was to minimize waste and biomass utilization of the juice production industry, to use fruit and berry by-products for pectin extraction with weak organic acid, and to determine the pectin yield and the physical, chemical, antioxidant and rheological properties of extracted pectin.

## 2. Materials and Methods

By-products (BP) of rhubarb (*Rheum rhabarbarum*), apples (*Malus domestica*), plums (*Prunus domestica*), red currants (*Ribes rubrum*), black currants (*Ribes nigrum*), gooseberries (*Ribes uva-crispa*), and sour cherries (*Prunus cerasus*) for the research were obtained from local juice and cider producers in Latvia, from June to August, 2022. Pumpkin (*Cucurbita pepo* L., pink banana jumbo) BP–peel and pomace were obtained from Lat Eko Food, Rudolfs, Ltd., Adazi, Latvia, September 2021. The BP were stored at T = −20 ± 2 °C in plastic bags until extraction and experiments.

### 2.1. Characterization of by-Products and Sample Preparation and Extraction

The BP were dispersed with 0.1 M citric acid (1:2 (*w*/*v*); pH = 1–1.5), homogenized, and the extraction of pectin was carried out according to the Citric Acid Method (CA) with some modifications [12,65]. The prepared samples were heated at 90 °C for 60 min, cooled and centrifuged (Sigma 4–16 KS, Germany) at 6000 rpm for 20 min. Collected supernatants were treated with absolute ethanol (1:2 (*v*/*v*)) at +4 ± 1 °C for 14–16 h. The pectin precipitates were collected by re-centrifugation, and then were washed twice with 80% (*v*/*v*), and 90% (*v*/*v*) ethanol, and the obtained pectin was conventionally hot–air dried (T = 40 ± 2 °C; t = 12–14 h) to a constant moisture content ≤ 9%.

### 2.2. Pectin Yield, Ash Content and Moisture Content

The pectin yield (DM) was calculated (Equation (1)).

(1) $$PY=\frac{m_{1}}{m_{2}}\times 100$$

PY—pectin yield, m_1_—weight of dried pectin, (g), m_2_—weight of by-product, (g), dry matter (DM).

The ash content in the extracted, dried pectin samples was determined according to the AOAC 942.05 method [66,67]. Moisture content (%) of by-products and resulting pectin samples was determined according to Reference method ISO 712:2009(EN).

### 2.3. Equivalent Weight, Methoxyl Content, Degree of Esterification

The determination of the pectin’s equivalent weight (EW) was carried out and calculated (Equation (2)) with the method described by Virk and Sogi (2007,) with some minor modifications [62]. The pectin (0.2 ± 0.02 g), ethanol (5 mL), sodium chloride (1.0 g), distilled water (100 mL), and 5–6 drops of phenol red indicator were dissolved and titrated against 0.1 N NaOH until the colour of the indicator changed (pH 7.5) to pink and persisted for at least 30 s.

(2) $$EW=\frac{m\times 100}{0.1\times V_{1}}$$

EW—equivalent weight, g mol^−1^; m—weight of pectin sample, g; 0.1—normality of alkali; V_1_—volume of alkali, mL.

The methoxyl content (ME) was determined using pectin saponification and titration of the liberated carboxyl groups. The neutralized solution obtained during the determination of equivalent weight was collected, and 25 mL of 0.25 N NaOH was added. The mixture was stirred thoroughly and kept at ambient temperature for 20 min. Then, 25 mL of 0.25 N HCl was added and titrated against 0.1 N NaOH to the end point. The calculation (Equation (3)) of ME value:

(3) $$ME,\%=\frac{3.1\times V_{2}\times N_{A}}{m}$$

ME—methoxyl content,%; V_2_—volume of alkali, mL; N_A_—normality of alkali; m—weight of pectin sample, g [13].

The degree of esterification (DE) was calculated (Equation (4)):

(4) $$DE,\%=100\times \frac{V_{2}}{\left(V_{1}+V_{2}\right)}$$

### 2.4. Fourier-Transform Infrared Spectroscopy (FT-IR) of Pectin Samples

The chemical structure of pectin extracted from BP was characterized using Fourier-Transform Infrared Spectroscopy (FT-IR) with a Nicolet 6700 FT-IR Spectrometer from Thermo Fischer (Waltham, MA, USA) for measuring all IR frequencies, Spectral Range (Standard) 7800–350 cm^−1^, Wavenumber Precision 0.01 cm^−1^.

### 2.5. Monosaccharide Composition and Galacturonic Acid Content

The monosaccharide composition of extracted pectin samples was analysed using methylation, followed by high-pH anion exchange chromatography with pulsed amperometry detection (HPAEC-PAD) and GC-MS analysis (triplicate runs) [68,69].

Samples (1.00 ± 0.02 mg) of dried pectin were mixed with 2 M HCl in methanol and flushed with argon. Methanolysis was then performed at 100 °C for 4 h. Samples were then neutralized with pyridine and dried under compressed air. Dried samples were subsequently hydrolyzed using 2 M trifluoracetic acid at 120 °C for 1 h, dried under compressed air and re-suspended in water. Hydrolysed monosaccharides were analyzed using HPAEC-PAD on an ICS6000 system (Dionex, Sunnyvale, CA, USA) using a Dionex CarboPac PA20 column, at 30 °C at a flow rate of 0.4 mL min^−1^.

### 2.6. Content of Total Phenolic Compounds, Antiradical Scavenging Activity (DPPH Method), Quantification of Phenolic Acids by HPLC

The content of total phenolic compounds (TPC) of the pectin extracts was determined according to the Folin–Ciocalteu Method, with some modifications [70,71,72,73,74,75].

A total of 20 ± 0.1 mg of the pectin sample was extracted with acetone, ethanol and water solution (7:7:6) in a volumetric 100 mL flask, in US bath for 10 min; 2.5 mL of Folin–Ciocalteu reagent (diluted 10 times with water) was added to 0.5 mL of the extracted sample in test tubes, and after 3 min 2.0 mL sodium carbonate (Na_2_CO_3_) solution (7.5%) was added. The resulting solution was mixed and allowed to stand for 30 min at 20 ± 1 °C in a dark place. Absorption was read at 765 nm, with a JENWAY 630 Spectrophotometer. Gallic acid (0–100 µg mL^−1^) was used for calibration of a standard curve. The results were expressed as the gallic acid equivalent of dry weight (µg GAE g^−1^ DW). Quantification was based on a standard curve (Equation (5)).

y = 0.0313 x + 0.055, R^2^ = 0.999 (5)

y—total content of phenolics, TPC, mg GAE 100 g^−1^, x—absorption at *λ* = 765 nm, R—coefficient of determination.

The antiradical scavenging activity of the pectin samples was determined using the method, based on the scavenging activities of the stable 2,2-diphenyl-1-picrylhydrazyl (DPPH) radical. The DPPH reagent (4 mg) was dissolved in MeOH (100 mL) for a solution concentration of 40 µL mL^−1^. To determine the scavenging activity, 100 µL DPPH reagent was mixed with 100 µL of sample extract in a test tube, and was incubated at room temperature for 30 min. After incubation, the absorbance was measured at *λ* = 514 nm. The antiradical scavenging activity of the pectin material was expressed (Equation (6)):

(6) $$A=\frac{AbsC-AbsS}{AbsC}\times 100$$

A—antiradical scavenging activity, AbsC—absorbance of control sample, AbsS—absorbance of pectin sample extract [39,40,41].

Free and total phenolic acids were quantified by saponification with high-pressure liquid chromatography (HPLC), Agilent Technologies 1200 Series, at 25.5–23.6 °C, 20 µL, flow 1 mL min, Max P 400 bar. Bound phenolic acids were liberated using 2 M NaOH (3 °C overnight in the dark, under stirring conditions), and then acid hydrolysis (12 M HCl to pH 2) was carried out, followed by extraction with ethyl acetate (1:1) 4 times, dried and redissolved with 10% acetic acid and MeOH mixture. All fractions were quantified separately, using HPLC. After HPLC quantification, the results of alkali and acid hydrolysates were calculated, to represent the total phenolic acids [76].

Internal standards of gallic, benzoic, caffeic, syringic, coumaric, ferrulic, sinapic, and cinnamic acids were used for quantification on a standard curve of phenolic acids in pectin samples, using the regression equations for each standard, R^2^ ≥ 0.999.

Folin–Ciocalteu′s Phenol reagent, Sigma-Aldrich, Cat. No. F9252, Sodium carbonate, Merck, Cat No. 106392, Gallic acid monohydrate (3,4,5-Trihydroxybenzoic acid monohydrate), ACS reagent, ≥98.0% Sigma-Aldrich, CAS Number 5995-86-8 2,2-diphenyl-1-picrylhydrazyl reagent—Sigma-Aldrich, CAS Number 1898-66-4, were used for TPC and antiradical scavenging activity determination.

### 2.7. Rheology of Pectin Gel Samples

The ability to form a stable gel was measured for the formulated solution of citric acid (7 ± 1%), sucrose (29 ± 1%), pectin (6 ± 1%) and water (58 ± 1%), for all samples [77]. Solutions of pectin/sucrose/citric acid were diluted in distilled water, mixed and heated at 95 °C for 10 min; afterwards, they were cooled and stored at +4 °C until the rheological measurement.

Rheological properties of the gels from pectin were determined with Anton Paar Modular Compact Rheometer, Smart Pave 102, (the cone and plate), Germany. *G*′ (the storage modulus—the elastic component of the material) and *G*″ (the loss modulus), the viscosity, 𝜂, and the loss factor, tan (*δ*) = *G*″/*G*′ were determined with a shear rate from 0.1 to 100 s^−1^ at 25 °C, in the linear range [77,78].

Apple pectin (Merk, Sigma Aldrich, Germany, CAS Nr. 9000-69-5) was used to compare physical, chemical and rheological properties.

All measurements were carried out for three independent samples (n = 3) and the results were expressed as mean values ± standard deviation (SD). A mathematical analysis of the data was performed using MS Excel data analysis, a single-factor ANOVA, correlation and regression analysis were used. The hypotheses were tested using a *p*-value method, and the factors were evaluated as relevant if *p* < α = 0.05. In the analysis of variance, the Tukey and Friedman test was used to justify the differences in the results among the studied samples [79].

## 3. Results and Discussion

### 3.1. Extraction of Pectin and Pectin Yield

**Figure 1:**
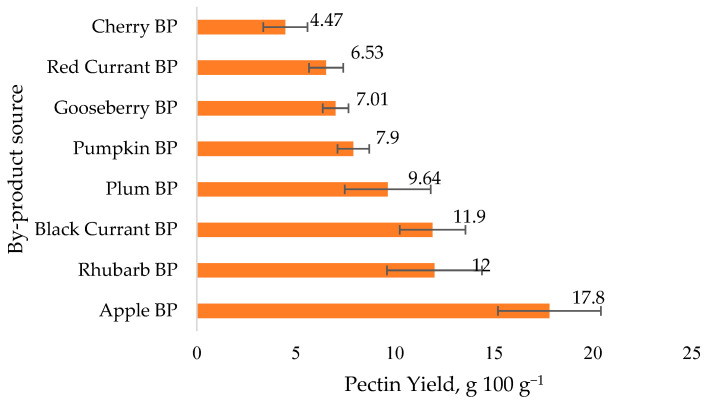
Pectin Yield from food production by-products with Citric Acid method. The data are presented as mean values (n = 3).

The yield of pectin in by-products (BP) is attached in Figure 1 and varied from 4.47 to 17.8%. The main properties of by-products are summarized in Table 1.

Many factors might influence the results of pectin yield in plant by-products: source of material, harvest time, pH of extracted solution, and time and temperature set for the experiment [3]. We also implemented some nonconventional extraction methods, resulting in a higher yield of pectin from pumpkin by-products, such as extraction with microwaves (>800 W) (16.0%) [14,16].

### 3.2. Ash and Moisture Content

The moisture content (Table 2) of extracted and conventionally dried pectin from by-products varied from 5.17 ± 0.12% to 6.12 ± 0.16%. Commercially produced pectin from apples has a moisture content ≤ 10%. Therefore, it can be considered that the water activity is decreased in the extracted pectin, and the growth of micro-organisms cannot further affect the pectin quality [16,80].

The ash content (Table 2) in the extracted pectin samples ranged from 1.42 ± 0.06% to 3.22 ± 0.21%. For good-quality gel formation from pectin, the maximum limit for ash content is 10% [81]. Therefore, the ash content in the research indicates the purity of the pectin from by-products.

### 3.3. Fourier-Transform Infrared Spectroscopy (FT-IR) of Pectin Samples

**Figure 2:**
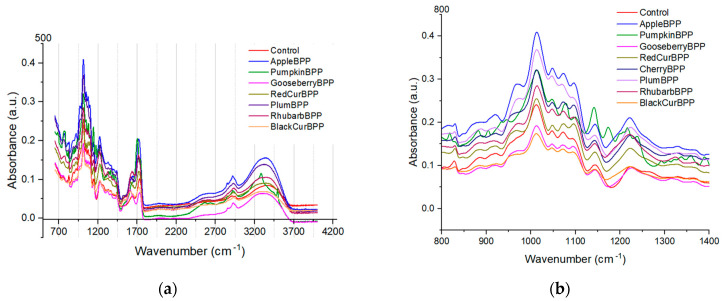
FT-IR spectra of the pectin from by-products and control sample from Sigma Aldrich Pectin; (**a**) FT-IR in the region 700–3700 cm^−1^; (**b**) FT-IR in the region 900–1400 cm^−1^.

The chemical structure of pectin extracted from various sources of by-products was characterized using FT-IR, and their spectra of typical chemical groups of pectin in the region 700 to 4000 cm^−1^ are presented in Figure 2 (Figure 2a) [13]. FT-IR spectra in the region between 800 and 1300 cm^−1^ (Figure 2b) are considered to form the ‘finger print’ region for carbohydrates, which allows an identification of major chemical groups specific to particular polysaccharides [12,81]. It can be observed that the samples extracted from by-products of berries, apples and pumpkin have spectra in the region similar to those of the control; therefore, the extracted polysaccharides obtained in this study were pectin. The region from 1400 cm^−1^ to 1800 cm^−1^ deals with information about functional groups occurring in molecules (Figure 2a); 1650 cm^−1^—Amid 1 C-N stretching, 1550 cm^−1^—Amid II N-H deformation [82,83]. Bands in the region 1760–1745 cm^−1^ are related to carbonyl groups, bands at 1640–1620 cm^−1^ are due to carboxylate ion stretching, and the band between 3000 and 3600 cm^−1^ is attributed to the stretching of O-H groups of the galacturonic acid polymer [2,12,18]. We detected peaks in this region for pumpkin BPP (3283 cm^−1^ and 3493 cm^−1^). There were no detected amide peaks related to proteins in the extracts.

### 3.4. Equivalent Weight, Methoxyl Content, Degree of Esterification

For pectin to be used in the food industry, it must meet certain quality criteria [3,83]. The equivalent weight (EW) is the most important physical property characteristic in determining the functional behavior of pectin [6,84]. The EW was significantly different among samples from different sources (*p* < 0.05). The equivalent weight (EW, g mol^−1^), (Table 3) in extracted pectin samples ranged from 417.2 ± 134.0 g mol^−1^ in rhubarb BPP to 1390 ± 469.0 g mol^−1^ in cherry BPP, compared with the control (Sigma Aldrich apple pectin) 708.5 ± 24.5 g mol^−1^. The EW values probably depend on the number of free acids, and the lower equivalent weight could be due to the partial degradation of pectin.

The degree of methyl esterification, DE (Table 3), is the main information about pectin properties required for many applications [27,85,86]. Many of the galacturonic acid residues have been esterified and, thereby, form methyl esters. Pectin with a degree of esterification, DE > 50%, is classified as high methoxyl (HM) pectin, and low methoxyl (LM) pectin has a DE < 50%. The obtained pectin from BP is HM pectin, as DE is >50%, except pectin from plums BP (DE = 45.16 ± 3.74%). LM pectin can be gelled with calcium ions and is independent of the presence of acid or a high solid content [6,87].

The pectin chemical structure undergoes important structural changes during the extraction procedures, and this results in a lower number of methylester groups [40]. The methoxyl content (ME)–the number of moles of methyl alcohol in 100 mol galacturonic acid in pectin–has an important role in determining the functional properties of pectin, including the structure and texture of the pectin gel formed, and in controlling the setting time [25].

The ME content in BPP (Table 3) ranged from 4.27 ± 1.04% in BPP for cherries to 8.13 ± 0.45% in BPP from black currants. Research into different pectin sources indicated an ME for banana pectin of 3.86–5.97, for citrus pectin 9.06 ± 0.03 and for apple pectin 7.92 ± 0.02 [12].

### 3.5. Monosaccharide Composition, Galacturonic Acid Content

The monosaccharide composition of pectin extracted using different methods and the control SA (Sigma Aldrich) is summarized (Supplementary Table S1). Glucose and galactose (from 3.89 ± 0.37 to 21.72 ± 1.95 g 100 g^−1^) were the main neutral sugar monosaccharides in the pectin samples, and their content was significantly higher in comparison with the control. Fucose (Fuc), rhamnose (Rha), and arabinose (Ara) were found in small amounts in the BPP, although the content of Ara and Rha was higher (1.58–6.30 g 100 g ^−1^ and 3.82–12.13 g 100 g^−1^, respectively), in comparison with the control. All BPP samples showed a high content of galactose. Other neutral monosaccharides, such as xylose and glucose, were also present, and were therefore assumed to be contaminants from the partial hydrothermal decomposition of hemicellulose [88]. Xyl content was lower in all BP pectin samples in comparison with the control.

Gal A content did not reach the target content of 65% in the apple BPP, plum BPP and rhubarb BPP samples. According to some researchers, the main parameter influencing the uronic acids content in the pectin extracts was the pH of the extraction mixture: with decreasing pH, the uronic acid content increased. In our study, the initial pH of the extraction mixtures had a relatively low value of 1.5–2.0 pH, and for further steps it is necessary to develop more sensitive methods for pectin extraction.

### 3.6. Content of Total Phenolics, Antiradical Scavenging Activity (DPPH Method)

The average content of total phenolic compounds (TPC, colorimetric method)) in pectin extracted from by-products is summarized in Table 4.

The highest content of TPC was in the black currant BPP (4.668 ± 0.405 µg GAE g^−1^) and in the plum BPP (3.102 ± 0.047 µg GAE, g^−1^).

**Figure 3:**
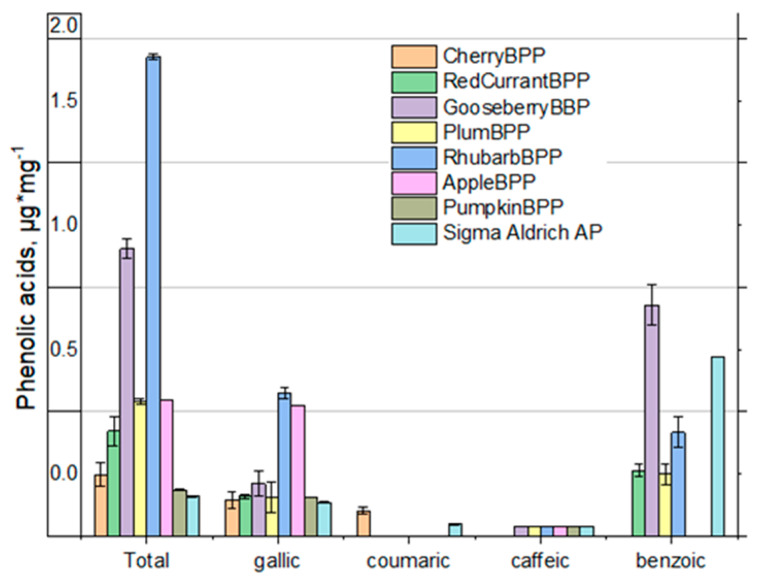
Phenolic acid profile of pectin samples (HPLC). Each pectin sample was prepared in three replicates and subsequently analyzed in triplicate.

The phenolic acid profile of eight selected phenolic acids—gallic, benzoic, caffeic, syringic, coumaric, ferrulic, sinapic, cinnamic acids—was analysed using HPLC, and these are summarized in Figure 3. Syringic, ferulic, and sinapic cinnamic acids were not detected in the pectin samples. Gallic acid and benzoic acid were of the highest content, with 0.57 µg mg^−1^ in the rhubarb BPP and 2.142 ± 1.660 µg mg^−1^ in the apple BPP.

The radical scavenging activity of pectin from different by-product sources was assessed against 2,2-diphenyl-1-picrylhydrazyl (DPPH) radicals, based on electron transfer, involving the reduction of a colored oxidant; all BPP samples showed high antiradical scavenging activity. Antiradical scavenging activity was 37.29 ± 2.03% in the black currant BPP.

Antiradical scavenging activity (DPPH method) was different in the pectin samples, and, furthermore, it was higher in comparison with the control sample. Strong correlation was found between TPC and DPPH (R^2^ = 0.887).

Antioxidant activity is important in the application of pectin to food products and biomedical products [87]. Depending on the number of aromatic rings, more than 8000 different phenolic compounds have been identified; moreover, phenolic compounds are reported to have a strong antioxidant activity, which can be associated with their ability to destroy oxygen-derived free radicals, break the radical chain reaction and/or chelate metals [88]. Although the determined number of phenolic acids is small in the pectin samples, their biological activity is very important. The biological activities of coumaric acid are antioxidant, antimicrobial and anti-inflammatory [89]. The anticancer properties of caffeic acid are related to its antioxidant and prooxidant capacity, and to its chemical structure containing free phenolic hydroxyls, moreover, these properties are also related to the ability to chelate metals such as copper (Cu) [90].

### 3.7. Rheology of Pectin Gel Samples

Methods for the determination of the rheological properties are necessary for the characterization of gels, as they contain a certain fraction of particles; moreover, the rheological behavior of these suspensions is important for a wide range of industrial, natural, and biological products and processes. Although more than 210 years have passed since the discovery of pectin, the chemical and structural properties are still under investigation, due to the heterogeneity of this family of polymers [3].

The methoxyl (ME) content and the anhydrouronic acid (AUA) levels, mainly galacturonic acid (GalA), in pectin, can affect the structure and texture of the pectin gel formed in the pectin samples [90,91]. HM pectin, with more than 50% of the methyl esterified carboxyl groups, forms physical gels at pH < 3.5 and in the presence of more than 55% (*w*/*v*) co-solutes, as sucrose; thus, an increased DE results in a more rapid gel formation [39,86].

The structure of gels depends on the interaction between pectin, sugar and acid. In our research, all the gel samples were prepared in equal concentrations of sucrose/pectin/citric acid. The gelation ability of gels containing pectin depends on the concentration and molecular weight of pectin—a higher molecular weight promotes gelling. The fruit and berries BPP contain natural carbohydrates, and there is no need to add them or add as much as is added by using commercial pectin in a powdered form. Gels from gooseberry BPP and apple pectin Sigma Aldrich formed stronger gel, and the damping factor or loss modulus at the gel stage was higher for those samples.

It is observed that viscosity is dependent on polymer chain length and also the degree of polymerization; diluting the polymer with too many additives may decrease viscosity and lead to a poor gelling ability [39,49]. Some researchers found that pectin with a higher viscosity can be obtained by using citric acid as solvent, and our study shows that high complex viscosity can be obtained with weak organic acid [12,85,86].

Pectin gels’ gelling ability is important for textural regulation in foods with pectin [92]. The strength of the gel is affected by the pH of the product, the pectin’s ME content, and the molecular weight [39,91]. Some studies reported that the type of acid influenced the macromolecular and gelling properties of the isolated pectin, and that citric acid was the least pectin-degrading, depolymerizing and de-esterifying extracting agent [93]. Low-DE pectin could also affect the viscosity of pectin solutions in further usage, and pectin with a lower DE could create a better condition for forming gels [92]. Gels from red currant BPP, apple BPP and gooseberry BPP were of higher viscous behavior, while, gels from Sigma Aldrich AP showed the lowest viscous behavior. The higher viscosity of pectin gels may be a result of smaller particles of the solid fraction [77,86,87,91].

**Figure 4:**
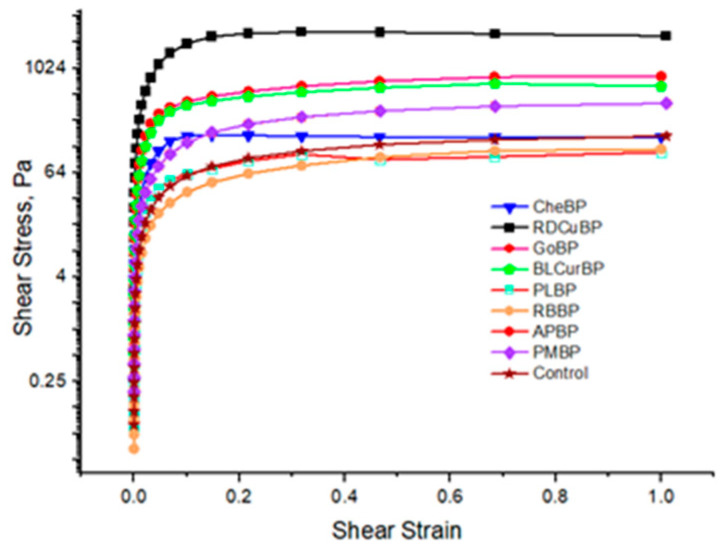
Shear stress of gels from BPP and Control (Sigma Aldrich AP).

Shear stress increased for all pectin gel samples with increasing shear strain, γ (rad) (Figure 4). The highest shear stress was observed for pectin gels from red currant BPP, gooseberry BPP and apple BPP, while the lowest shear stress was for rhubarb BPP; therefore, it should be considered that, in this case, gels will be formed with calcium ions, adding 6 mM CaCl [94]. Shear stress was significantly different (*p* > 0.05) for all pectin gel samples, in comparison with the control—Sigma Aldrich apple pectin.

**Figure 5:**
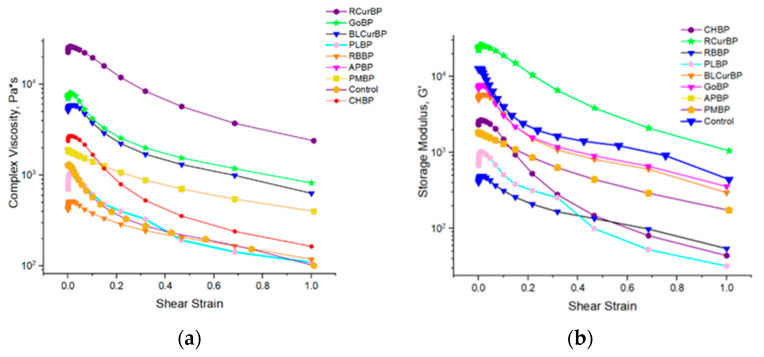

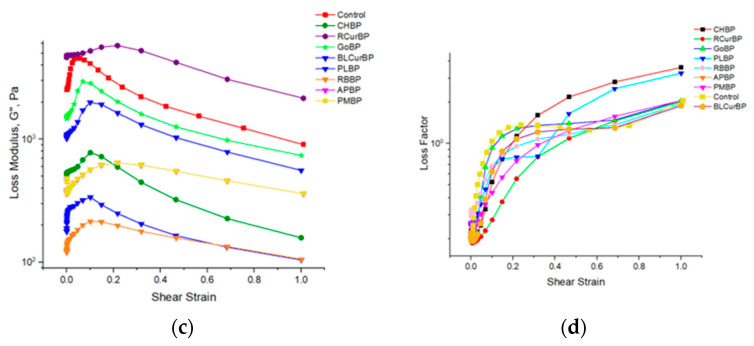
Rheological behavior of pectin gels: (**a**) complex viscosity of gels; (**b**) storage modulus of gels; (**c**) loss modulus of gels; (**d**) loss factor of pectin gels.

Storage modulus (G′, Pa)—the elasticity of pectin gels and the loss modulus (G″, Pa) were determined; the results of G′, G″—damping factor (tan δ, G″/G′) are presented (Figure 5). The complex viscosity (CV) (Figure 5a) for the BPP gels was different; the lowest CV was observed for the pectin gels from rhubarb BPP and plum BPP and for Sigma Aldrich AP; for all types of pectin gels, the CV was found to decrease with an increase in the shear strain, γ, and they demonstrated a better shear-thinning flow property than that of the control gel; the highest complex viscosity was observed for samples of red currant BPP gel. G′, the elasticity of gels from BPP, was higher (Figure 5b,c) in comparison with the control. 

Storage modulus or elasticity of gels from BPP remain more elastic; moreover, the gel from red currant BPP was of the highest elasticity (Figure 5b). Storage modulus (Figure 5b) decreased for all pectin gel samples. The viscous behavior of pectin gel samples from BP—loss modulus *G*″ (Figure 5c) was different from the samples with the control pectin; the gel samples from red currant BPP were of the highest loss modulus, followed by the gooseberry BPP gel and the apple BPP gel. It was observed that G′ *>* G″ in all pectin samples in the testing area, indicating that pectin gels from the extractions remained in the gel network. The ratio G′/G″ determines the condition for weak and strong gels [78]. 

With the increase in shear strain, the loss factor for all the pectin gel samples increased (Figure 5d), indicating, therefore, that the gels would change to liquid (damping factor > 1) when a higher angular frequency was applied [78]. The G′/G″ values of the pectin solutions were almost similar to those of the control from Sigma Aldrich. A loss factor of 1 means the substance is in transition between a liquid and a solid state, that is, the gel point [77,94].

## 4. Conclusions

By-products from rhubarb (*Rheum rhabarbarum*), apples (*Malus domestica*), plums (*Prunus domestica*), red currants (*Ribes rubrum*), black currants (*Ribes nigrum*), gooseberries (*Ribes uva-crispa*), sour cherries (*Prunus cerasus*) and pumpkin (*Cucurbita pepo)* are good starting materials for the extraction of pectin with high antioxidant activity. The highest pectin yields were obtained from apple by-products (BP), whereas the lowest results were from cherry BP and red currants BP. Most pectin from fruit by-products has a high degree of esterification and a high content of galacturonic acid. The extracted pectin from BP can be classified as high methoxyl (HM) pectin, since an exception occurred in the case of plums. The produced pectin gels in acidic conditions show good rheological properties—texture, gel stability and viscous behavior. The predominant monosaccharides in pectin samples are glucose and galactose, whereas the phenolic acid profile includes gallic acid and the minor content of benzoic, coumaric and caffeic acids. The extracted pectin can be used for functional products in the food industry and in cosmetics as a gelling or binding agent, due to its high content of phenolics and antioxidant activity. The high antioxidant activity of the food by-product’s pectin gives us reason to believe that this type of pectin could be further included in functional products with high bioactivity to promote innovative cosmetic and pharmaceutical products, as it contains phenolic acids, especially coumaric and caffeic acids. For further development of new products with pectin, more information about the behavior of such polysaccharides during the technological processes at a high temperature, is necessary.

## Figures and Tables

**Table 1:** Properties of by-products after juice extraction.

By-Product Source	Moisture,%	Ash, g 100 g^−1^ (DW)	Total Titratable Acidity (TA)
Cherry BP	63.2 ± 1.4	0.82 ± 0.2	4.25 ± 0.8
Red Currant BP	57.1 ± 0.9	1.24 ± 0.2	4.18 ± 0.2
Gooseberry BP	58.0 ± 1.3	0.92 ± 0.4	2.95 ± 0.1
Black Currant BP	55.4 ± 1.2	0.98 ± 0.3	4.22 ± 0.2
Plum BP	60.9 ± 0.8	0.76 ± 0.1	3.95 ± 0.3
Rhubarb BP	62.7 ± 2.4	0.62 ± 0.2	3.22 ± 0.1
Apple BP	64.9 ± 1.8	0.98 ± 0.1	3.65 ± 0.2

DW—Dry weight; Mean ± SD; the data are presented as mean values (n = 3).

**Table 2:** Properties of pectin obtained from juice production by-products.

Source	Moisture, %	Ash, g 100 g^−1^
Cherry BPP	6.0 ± 0.1	1.94 ± 0.05
Red Currant BPP	6.4 ± 0.1	1.42 ± 0.06
Gooseberry BPP	7.4 ± 0.2	2.24 ± 0.08
Black Currant BPP	6.2 ± 0.1	2.68 ± 0.02
Plum BPP	5.2 ± 0.1	2.11 ± 0.24
Rhubarb BPP	7.2± 0.2	2.14 ± 0.08
Apple BPP	6.5 ± 0.1	2.88 ± 0.14
Pumpkin BPP	7.2 ± 0.1	2.65 ± 0.18

Mean ± SD; the data are presented as mean values (n = 3).

**Table 3:** Properties of pectin obtained from juice extraction and primary food production by-products.

Source	DE, %	EW, g mol^−1^	ME, %
Cherry BPP	64.06 ± 3.83 ^b^	1390.0 ± 69.0 ^a^	4.27 ± 1.04 ^a^
Red currant BPP	57.12 ± 4.07 ^a^	716.9 ± 80.95 ^b^	6.35 ± 1.84 ^c^
Gooseberry BPP	57.14 ± 7.18 ^a^	895.8 ± 316.8 ^c^	5.53 ± 0.77 ^b^
Black currant BPP	54.69 ±2.67 ^a^	465.0 ± 72.04 ^d^	8.13 ± 0.45 ^e^
Plum BPP	45.16 ± 3.74 ^c^	531.6 ± 95.8 ^e,d^	5.15 ± 2.10 ^b^
Rhubarb BPP	54.32 ± 0.44 ^a^	417.2 ± 34.0 ^d^	5.52 ± 2.85 ^b^
Apple BPP	56.03 ± 3.03 ^a^	581.0 ± 46.1 ^e^	6.86 ± 0.77 ^c^
Pumpkin BPP	51.11 ± 0.69 ^a,c^	426.0 ± 8.7 ^d^	7.61 ± 0.30 ^d,e^
Control	56.69 ± 0.39 ^a^	708.5 ± 24.5 ^b^	5.73 ± 0.11 ^b,c^

BPP—by-product pectin; Control—apple pectin from Sigma Aldrich. Similar letters (a, b, c, d, e) indicate no significant difference among samples in column (*p* < 0.05). Mean ± SD; the data are presented as mean values (n = 3).

**Table 4:** The Total Phenolic Content, µg GAE mL^−1^ DW and Antiradical Scavenging activity (DPPH method) of the pectin extracts.

Source of Pectin	TPC, µg GAE g^−1^ DW	Antiradical Scavenging Activity, g 100 g ^−1^
Control	2.803± 0.002 ^b,c^	0.56 ± 0.04 ^e^
Apple BPP	2.398 ± 0.047 ^b^	2.27 ± 0.82 ^d^
Rhubarb BPP	2.173 ± 0.024 ^b^	8.79 ± 0.44 ^c,b^
Plums BPP	3.102 ± 0.047 ^c,b^	11.86 ± 1.12 ^b^
Red Currant BPP	2.516 ± 0.048 ^b^	7.57 ± 0.88 ^c^
Gooseberry BPP	2.657 ± 0.118 ^b^	15.97 ± 1.23 ^b^
Pumpkin BPP	2.076 ± 0.048 ^b^	3.03 ± 1.01 ^d^
Black Currant BPP	4.668 ± 0.405 ^a^	37.29 ± 2.03 ^a^

BPP—by-product pectin. Each pectin sample was analysed in triplicate. Similar letters (a, b, c, d, e) indicate no significant difference among samples in column (*p* > 0.05). Mean ± SD.

## Data Availability

Data is contained within the article or Supplementary Materials.

## Supplementary Materials

The following supporting information can be downloaded at: https://www.mdpi.com/article/10.3390/foods12081615/s1, Table S1: Monosaccharide and uronic acid content in pectin samples (g 100 g^−1^).
