# Supplementary material for: Pectin from Fruit- and Berry-Juice Production by-Products: Determination of Physicochemical, Antioxidant and Rheological Properties
Source: Foods. 2023 Apr 11;12(8):1615. doi: 10.3390/foods12081615 (PMC10137805; doi:10.3390/foods12081615)
Supplement: Supplementary file 1 [file foods-12-01615-s001.zip › foods-2244001-supplementary.pdf]

Manuscript-Supplementary **Table S1.**

Table S1. Monosaccharide and uronic acid content in pectin samples (g 100 g^{-1})

	Cherry BPP	Red Currant BPP	Gooseberry BPP	Black Currant BPP	Plum BPP	Rhu BPP	Apple BPP	Pumpkin BPP	Control
Fuc	0.14 ± 0.03	0.19 ± 0.02	0.11 ± 0.02	0.17 ± 0.02	0.08 ± 0.02	0.14 ± 0.02	0.22 ± 0.01	0.18 ± 0.01	0.13 ± 0.03
Rha	12.13 ± 0.70	4.82 ± 0.35	6.92 ± 0.20	5.56 ± 0.38	5.55 ± 0.96	3.82 ± 0.74	10.12 ± 0.62	5.06 ± 1.29	3.81 ± 0.97
Ara	2.94 ± 0.90	1.76 ± 0.09	2.70 ± 0.31	1.58 ± 0.09	3.10 ± 0.99	6.30 ± 0.45	2.68 ± 0.36	2.53 ± 1.51	1.40 ± 0.07
Gal	6.20 ± 1.82^{b}	3.89 ± 0.37^{d}	9.04 ± 0.44$^{b\ c}$	11.91 ± 2.23^{c}	21.72 ± 1.95^{a}	20.58 ± 1.37^{a}	4.28 ± 0.41^{e}	8.35 ± 0.83^{b}	7.42 ± 0.89^{b}
Glu	15.0 ± 2.82^{b}	4.57 ± 0.26^{c}	5.84 ± 0.10^{c}	8.63 ± 1.61^{d}	23.40 ± 2.43^{a}	12.14 ± 1.38^{b}	22.95 ± 2.95^{a}	6.92 ± 0.47$^{c\ d}$	10.38 ± 1.54^{b}
Xyl	0.36 ± 0.02	0.31 ± 0.07	0.22 ± 0.00	0.56 ± 0.13	0.58 ± 0.13	0.50 ± 0.11	1.32 ± 0.19	0.45 ± 0.01	2.13 ± 0.41
Man	0.57 ± 0.03	0.14 ± 0.01	0.13 ± 0.02	0.55 ± 0.05	0.31 ± 0.12	0.17 ± 0.08	0.08 ± 0.02	0.41 ± 0.04	0.14 ± 0.08
4O-MeGlcA	0.22 ± 0.14	0.49 ± 0.22	0.05 ± 0.06	0.57 ± 0.16	0.07 ± 0.10	0.06 ± 0.01	0.16 ± 0.09	n.d.	0.20 ± 0.02
GalA	59.06 ± 9.79^{a}	83.57 ± 0.56^{c}	74.88 ± 0.42^{b}	69.16 ± 4.00$^{b\ a}$	47.22 ± 3.63^{d}	55.77 ± 0.60^{a}	57.80 ± 2.45^{a}	75.34 ± 0.04^{b}	74.04 ± 3.73^{b}
GlcA	0.09 ± 0.02	0.10 ± 0.01	0.13 ± 0.09	0.21 ± 0.09	0.38 ± 0.07	0.52 ± 0.08	0.39 ± 0.09	0.76 ± 0.16	0.13 ± 0.01

BPP – by-product pectin, n.d. – not detected. Each pectin sample was prepared and subsequently analysed in triplicate (n = 3), ± standard deviation (SD). Values superscripted in rows with different letters (a, b, c, d, e) are statistically different ($p< 0.05$). Fuc (fucose, g 100 g^{-1}); Rha (rhamnose, g 100 g^{-1}); Ara (arabinose, g 100 g^{-1}); Gal (galactose g 100 g^{-1}); Glu (glucose, g 100 g^{-1}); Xyl (xylose, g 100 g^{-1}); Man (mannose, g 100 g^{-1}).
